# The presence of antibiotic-resistant bacteria at four Norwegian wastewater treatment plants: seasonal and wastewater-source effects

**DOI:** 10.3389/frabi.2024.1351999

**Published:** 2024-02-07

**Authors:** Daniel Basiry, Roald Kommedal, Krista Michelle Kaster

**Affiliations:** Department of Chemistry, Bioscience and Environmental Engineering, Faculty of Science and Technology, University of Stavanger, Stavanger, Norway

**Keywords:** wastewater treatment plant, sequencing, antibiotic, antibiotic resistance genes, seasonal variation, Norway, microbial community effects

## Abstract

Wastewater treatment plants receive low concentrations of antibiotics. Residual concentrations of antibiotics in the effluent may accelerate the development of antibiotic resistance in the receiving environments. Monitoring of antimicrobial resistance genes (ARGs) in countries with strict regulation of antibiotic use is important in gaining knowledge of how effective these policies are in preventing the emergence of ARGs or whether other strategies are required, for example, at-source treatment of hospital effluents. This study evaluates the presence of certain common resistance genes (*bla*_SHV-1_, *bla*_TEM-1_, *msrA*, *ermA*, *ermC*, *tetM*, *tetL*, *tetA*, *vanA*, and *vanC*) in the influent, sludge, and effluent of four wastewater treatment plants (WWTPs) in the North Jæren region of Norway at two different sampling times (January and May). These WWTPs vary in drainage area and wastewater composition and were selected based on their differing wastewater characteristics. Randomly selected colonies from the activated sludge samples were used to determine the minimum inhibitory concentration (MIC) for ampicillin, vancomycin, and tetracycline. In addition, variations in the bacterial composition of the wastewater were characterized via 16S rRNA sequencing and were analyzed in terms of bacterial host taxa that explain the presence of the ARGs in wastewater. The MIC tests revealed MIC_90_ values of >128 µg/mL for ampicillin, ≥128 µg/mL for vancomycin, and 32 µg/mL for tetracycline. In addition, the three resistance genes, *ermB*, *tetA*, and *tetM*, that were present in the influent and activated sludge were still present in the effluent. These results indicate that WWTPs represent a direct route into the environment for resistance genes and do not significantly reduce their abundance. Hence, the development of treatment methods for the removal of these genes from WWTPs in the future is of utmost importance.

## 1 Introduction

The rapidly increasing global development of antimicrobial resistance (AMR) represents a threat to health worldwide. It is predicted that antibiotic-resistant bacteria (ARBs) will become one of the leading causes of death and their development will lead to major financial losses, especially in low- to middle-income countries (Morrison and Zembower, 2014; Durand et al., 2019). Identifying and limiting probable locations for the development and distribution of ARBs is pivotal in gaining understanding of them and in the development of new strategies and policies to help prevent further resistance development. Wastewater treatment plants (WWTPs) have been identified as hotspots for and distributors into the environment of antibiotic resistance genes (ARGs) (Karkman et al., 2018; Pazda et al., 2019). When humans or animals ingest antibiotics, large amounts are excreted intact *via* urine and can enter the sewer system (Kümmerer, 2009). Gut bacteria from humans and animals are a major source of ARGs (Kent et al., 2020). Hence, it is very likely that WWTPs receive a constant stream of low levels of both antibiotics and ARBs. WWTP design has focused on the removal of organic matter, nitrogen, and phosphorous from the influent, with the removal of ARGs and ARBs as a lower priority (Pruden, 2014).

Wastewater is rich in nutrients (Morée et al., 2013), contains a diverse and dense microbial community (Qin et al., 2018), and often contains low, sublethal doses of antibiotics (Chow et al., 2021), rendering it a good environment for the transfer of ARGs. Antibiotic concentrations below the minimum inhibitory concentration (MIC) are known to lead to selectivity for ARGs. The minimal selective concentration (MSC) can be much lower than the MIC. Gullberg et al. (2011) found that the MSC values of various antibiotics could be anywhere from 1/4 to 1/230 of the MIC. In addition, exposure to antibiotics can result in increased mutation rates due to the SOS response (Chow et al., 2021). ARGs can be transferred between species by horizontal gene transfer (HGT) mechanisms such as integrons, plasmids, and transposons (Abby et al., 2012; Redondo-Salvo et al., 2020). It has been shown in the case of the human gut microbiota that HGT can play a major role in the spread of ARGs (Kent et al., 2020; Li et al., 2020).

Recently, the use of sewage systems for monitoring of ARGs has garnered interest (Kwak et al., 2015; Hutinel et al., 2019; Huijbers et al., 2020). By investigating the microbial communities and the distribution of ARGs present in wastewater, the probable abundance and types of ARBs that may be circulating in that area may be determined. Norway has relatively low antibiotic consumption and abundance of ARBs (World Health Organization, 2018), and has decreased its consumption of antibiotics further below the European average in 2021 (European Centre for Disease Prevention and Control (ECDC), 2021). Norway therefore represents a good example of a country on the lower end of antibiotic consumption (World Health Organization, 2018). To determine whether decreased antibiotic consumption affects the ARBs present in WWTPs, it is important to investigate the antimicrobial resistance of genes and bacteria circulating in Norwegian WWTPs. This study aimed to evaluate the presence of ARGs of concern (*bla*_SHV-1_, *bla*_TEM-1_, *msrA*, *ermA*, *ermC*, *tetA*, *tetL*, *tetM*, *vanA*, and *vanC*) in the inlet, the sludge produced, and the effluent of four Norwegian WWTPs with differing wastewater characteristics, catchment area activities, and process configurations. To evaluate seasonal changes in the abundance of ARGs and ARBs, two contrasting months, one in the winter and one in the spring, were selected for sampling. In addition, the bacterial community composition was sequenced for detailed mapping of the microorganisms present and to assess changes in the microbial communities. Finally, random colonies were isolated, and minimum inhibitory concentration tests for ampicillin, tetracycline, and vancomycin were conducted to understand the dynamics of resistance to popular antibiotics.

## 2 Methods

### 2.1 Study sites and sample collection

Grab samples were obtained from four different WWTPs [Vik RA, Bore, Grødaland, and Mekjarvik (Sentralrenseanlegg Nord-Jæren [SNJ])] in the North Jæren region of southwest Norway. One-liter samples were taken from the wastewater inlet and outlet and from the produced sludge at two separate sampling times in January and May 2022. These two months were selected to represent the beginning and end of the winter flu season. The WWTPs were selected to reflect variability in wastewater composition, mass loadings, industrial to municipal wastewater fraction, and treatment process configuration. Of particular interest was the ratio of agro-industrial to municipal household loading. Mekjarvik (SNJ) represents typical urban municipality wastewater; Vik RA, mixed municipal and food industry wastewater; Grødaland, agro- and food industry-dominated wastewater; and Bore, a typical rural municipal sewage treatment plant. Refer to **Supplementary Table S1** for details and **Supplementary Figure S1** for the locations. Samples were stored in cool bags and transported by car to the laboratory immediately after sampling; biological and analytical tests were conducted immediately upon arrival at the laboratory.

### 2.2 Wastewater compositional analysis

Total suspended solids (TSS) was measured following the standard method 2540D (Lipps et al., 2022) using duplicate filtered samples (preweighed glass fiber filter, pore size 0.45 μm, diameter 47 mm, 934-AH RTU; Whatman, Kent, UK), which were dried at 105°C for 1 h and weighed analytically (XP205, Mettler Toledo, Switzerland). Volatile suspended solids (VSS) was determined following the standard method 2540E using the same filters as for the TSS analysis, with the retained particles combusted at 550°C for 30 minutes (Rice et al., 2013).

### 2.3 Isolation of strains

Strains were isolated for the MIC tests by diluting 1 g of centrifuged activated sludge in 99 mL Mueller–Hinton broth. A 1:10 serial dilution series was then spread on Mueller–Hinton agar plates and incubated overnight at room temperature (20°C–25°C) (as the WWTPs were not operated at 37°C). The procedure was adapted with modifications from Reddy et al. (1986). As not all colonies could be tested, the next morning, 24 colonies were randomly picked and grown in liquid Mueller–Hinton broth, and a cryostock for each of the isolated strains was stored in a freezer at −80°C.

### 2.4 Antibiotic susceptibility testing

The MICs of antibiotics for the 24 bacterial isolates from each sludge sample were determined by a standard twofold serial broth dilution method using Mueller–Hinton broth in 96-well plates (Greiner Bio-One, Kremsmünster, Austria). This procedure was conducted according to the international standard ISO 20776-1:2019 (212, T. C. I, 2019) and Wiegand et al. (2008) using antibiotic concentrations ranging from 0.25 to 128 mg/L. The inoculum was grown to a turbidity of 0.5 McFarland standard, measured using a photometer (Genesys 50; Thermo Fisher Scientific, Waltham, MA, USA). The 96-well plates were then inoculated and incubated for 20 h at 25°C. In this study, three antibiotics were tested: ampicillin (AMP) (Sigma-Aldrich, St. Louis, MO, USA), tetracycline (TET) (Sigma-Aldrich), and vancomycin (VAN) (Millipore, Billerica, MA, USA). Ampicillin was included as it is extensively used in human and veterinary medicine (Klein et al., 2021); tetracycline, on the basis that it is one of the major antimicrobials used in animal husbandry (Rushton et al., 2014); and vancomycin, as it is one important treatment for methicillin-resistant *Staphylococcus aureus* (Shariati et al., 2020). In addition to the varying antibiotic concentrations, growth was verified via positive and negative wells, which contained no antibiotic and no bacterial isolate, respectively.

### 2.5 DNA extraction and PCR

DNA was extracted from activated sludge and water samples upon arrival at the laboratory. For each sample, 50 mL activated sludge was taken and centrifuged at 5,000 *g* for 10 minutes (Falcon tube), and 0.3 mg of the pellet was used for DNA extraction. Subsequently, 50 mL water samples were filtered through autoclaved OE66 membrane filters (cellulose acetate, pore size 0.2 μm, diameter 47 mm; Whatman, UK) cut into small pieces. DNA extraction was then conducted using the DNeasy PowerSoil Pro Kit (QIAGEN, Hilden, Germany), following the manufacturer’s instructions. A NanoVue™ Plus Spectrophotometer (GE Healthcare, Chicago, IL, USA) was used to quantify the extracted DNA and ensure the quality of DNA using an A_260/280_ measurement of approximately 1.8.

A 20-µL final volume was used for polymerase chain reaction (PCR) amplification. The PCR mix contained 2 µL 10x Key Buffer Mg^2+^ free (VWR, Radnor, PA, USA), 1 µL dNTP, 0.3 µL of each primer (20 µM), *Taq* DNA Polymerase (Invitrogen, Carlsbad, CA, USA), 1 µL template DNA, and 15.7 µL molecular-grade water (Mediatech, Inc., Manassas, VA, USA). A 2720 thermal cycler (Applied Biosystems, Foster City, CA, USA) was used to amplify the targeted sequences. The sequences of the PCR primers and the amplification cycles used are presented in **Table 1**. All samples from the four WWTPs were tested for resistance genes with positive and negative controls.

**Table 1:** Primers used for various antibiotic resistance genes and their annealing temperatures.

Gene	Orientation	Sequence (5′–3′)	Annealing	Reference
***bla*_SHV-1_**	Forward	TCAGCGAAAAACACCTTG	57°C	(Dehshiri et al., 2018)
	Reverse	TCCCGCAGATAAATCACC		
***bla*_TEM-1_**	Forward	CATTTCCGTGTCGCCCTTAT	55°C	(Randall et al., 2004)
	Reverse	TCCATAGTTGCCTGACTCCC		
***msrA***	Forward	TCCAATCATTGCACAAAATC	55°C	(Martineau et al., 2000)
	Reverse	AATTCCCTCTATTTGGTGGT		
***ermA***	Forward	TATCTTATCGTTGAGAAGGGATT	55°C	(Martineau et al., 2000)
	Reverse	CTACACTTGGCTTAGGATGAAA		
***ermB***	Forward	AAAAATATAAAATATTCTCA	49°C	(Piotrowska et al., 2017)
	Reverse	TAGACAATACTTGCTCATAAGTAAC		
***ermC***	Forward	CTTGTTGATCACGATAATTTCC	55°C	(Martineau et al., 2000)
	Reverse	ATCTTTTAGCAAACCCGTATT		
***tetA***	Forward	GCTACATCCTGCTTGCCTTC	55°C	(Ng et al., 2001)
	Reverse	CATAGATCGCCGTGAAGAGG		
***tetL***	Forward	TTTCGGGTCGGTAATTGG	55°C	(Fiedler et al., 2016)
	Reverse	GGCTATCATTCCACCAATCG		
***tetM***	Forward	ACCTGAGCAATGGGATGTGG	55°C	(Fiedler et al., 2016)
	Reverse	GCTGCTCAATCCCTATGTTGC		
***vanA***	Forward	AATGTGCGAAAAACCTTGCG	58°C	(Lu et al., 2001)
	Reverse	CCGTTTCCTGTATCCGTCC		
***vanC***	Forward	GATGGCWGTATCCAAGGA	57°C	(Patel et al., 1997)
	Reverse	GTGATCGTGGCGCTG		

The genes were selected based on common resistance genes present in WWTPs and after reviewing the literature (Özkök, 2012; Jang et al., 2018; Hubeny et al., 2019; Pallares-Vega et al., 2019; Peirano and Pitout, 2019; Mirkalantari et al., 2020). **Table 2** shows the corresponding gene family and the drugs they work against.

**Table 2:** Antibiotic resistance genes, their gene families, and the target antibiotic classes against which resistance is conveyed.

	Gene family	Drug class
***bla*_SHV-1_**	β-Lactamase	Penam, monobactam, cephalosporin
***bla*_TEM-1_**	β-Lactamase	Carbapenem, penam, cephalosporin
***msrA***	msr-Type ABC-F ribosomal protection protein	Streptogramin B, macrolide
***ermA***	Erm 23S ribosomal RNA methyltransferase	Streptogramin, lincosamide, macrolide
***ermB***	Erm 23S ribosomal RNA methyltransferase	Streptogramin, lincosamide, macrolide
***ermC***	Erm 23S ribosomal RNA methyltransferase	Streptogramin, lincosamide, macrolide
***tetA***	Major facilitator superfamily (MFS) efflux pump	Tetracycline
***tetL***	MFS efflux pump	Tetracycline
***tetM***	Tetracycline-resistant ribosomal protection protein	Tetracycline
***vanA***	Van ligase	Glycopeptide
***vanC***	Van ligase	Glycopeptide

### 2.6 Sequencing

Extracted DNA from the inlet, activated sludge, and effluent was normalized to ~100 ng/µL, frozen at −20°C until all samples had been collected, and subsequently submitted to Novogene (UK) Company Limited (Cambridge, UK) for external sequencing.

The V3–V4 region of the 16S rRNA gene for bacterial DNA was targeted using the B-341F (5′-CCTAYGGGRBGCASCAG) and B-806R (5′-GGACTACNNGGGTATCTAAT) primers amplifying 470 base pairs (bp). The library was created by “sequencing by synthesis” using the Illumina NovaSeq 6000 platform, and 250-bp conducted paired-end reads were then created by Novogene. Raw reads were assigned to samples and merged using the FLASH (fast length adjustment of short reads) software. The clean tags were clustered into operational taxonomic units (OTUs) with 97% similarity (Youssef et al., 2009; Caporaso et al., 2011; Hess et al., 2011). The sequencing data can also be found in the European Nucleotide Archive (accession number PRJEB70859).

## 3 Results

### 3.1 Minimum inhibitory concentration of strains

The antibiotic resistance levels for tested bacteria obtained from randomly selected colonies from the 16 sludge samples, displayed as MIC_50_ and MIC_90_, are presented in **Table 3**. MIC_50_ and MIC_90_ respectively represent the MIC values at which 50% and 90% of the isolates were inhibited by the administered antibiotic concentrations. The individual values for each isolate can be found in **Table S3**.

**Table 3:** MIC_50_ and MIC_90_ values for the antibiotics ampicillin, vancomycin, and tetracycline in mg/L, representing the MIC value that includes 50% or 90% of the samples, respectively.

		January		May	
	Range	MIC_50_	MIC_90_	MIC_50_	MIC_90_
Ampicillin					
Vik RA	0.25–128	>128	>128	>128	>128
Bore	0.25–128	>128	>128	>128	>128
Grødaland	0.25–128	>128	>128	64	>128
Mekjarvik (SNJ)	0.25–128	>128	>128	64	>128
Vancomycin					
Vik RA	0.25–128	32	128	128	128
Bore	0.25–128	32	128	128	>128
Grødaland	0.25–128	64	>128	64	>128
Mekjarvik (SNJ)	0.25–128	32	>128	64	>128
Tetracycline					
Vik RA	0.25–128	0.5	4	1	16
Bore	0.25–128	1	32	4	8
Grødaland	0.25–128	1	32	1	4
Mekjarvik (SNJ)	0.25–128	1	8	1	16

The MIC_50_ and MIC_90_ values for ampicillin were above the measured concentration range of 128 µg/mL in all WWTPs in January. The same result was observed in May for the Vik RA and Bore WWPTs. For the other two WWTPs, the MIC_50_ values decreased to 64 µg/mL in May, exhibiting a reduction in overall ampicillin resistance.

In general, an increase in vancomycin susceptibility could be observed from January to May. The MIC_90_ for vancomycin remained the same from January to May except in Bore, where it increased from 128 µg/mL to above the tested values. The MIC_50_ values increased in Vik RA and Bore to 128 µg/mL in May, and to 64 µg/mL in Mekjarvik (SNJ). The WWTPs in Vik RA and Mekjarvik (SNJ) had higher MIC_90_ values in May. In Bore and Grødaland, a reduction in the top 10% occurred.

The MIC_50_ and MIC_90_ values for tetracycline were far lower than those for ampicillin and vancomycin at both sampling times and at all four WWTPs: the MIC_50_ values were 1 µg/mL in January and May, with the exception of Vik RA in January, where the value was 0.5 µg/mL, and Bore in May, with 4 µg/mL. There was more variability in values of MIC_90_. In January, Bore and Grødaland had the highest values at 32 µg/mL, whereas Vik RA had the lowest at 4 µg/mL. In May, the highest values were in Vik RA and Mekjarvik (SNJ) at 16 µg/mL, whereas the lowest was in Grødaland at 4 µg/mL.

### 3.2 Antibiotic resistance genes

The presence or absence of the resistance genes *bla*_SHV-1_, *bla*_TEM-1_, *msrA*, *ermA*, *ermC*, *tetA*, *tetL*, *tetM*, *vanA*, and *vanC* was examined via PCR testing; the results are presented in **Table 4**. The majority of the resistance genes were absent from all samples tested. However, *ermB*, *tetA*, and *tetM* were found in all samples, with the exceptions of the outlet samples taken from Vik RA and Mekjarvik (SNJ) in May. In Vik RA, none of the three resistance genes (*ermB*, *tetA*, and *tetM*) were found. In Mekjarvik (SNJ), only *ermB* was present. The *msrA* resistance gene was found in the samples taken from Bore in May and from Grødaland in both January and May, except in the case of the sludge sample taken in January, where the *msrA* gene was not found. Common resistance genes such as *bla*_TEM-1_ were not found in any of the samples.

**Table 4:** The presence of different antibiotic resistance genes at the WWTPs in January and May, as tested *via* PCR.

	*bla*_SHV-1_	*bla*_TEM-1_	*msrA*	*ermA*	*ermB*	*ermC*	*tetA*	*tetL*	*tetM*	*vanA*	*vanC*
**BIJ**	**−**	**−**	**−**	**−**	**+**	**−**	**+**	**−**	**+**	**−**	**−**
**BSJ**	**−**	**−**	**−**	**−**	**+**	**−**	**+**	**−**	**+**	**−**	**−**
**BOJ**	**−**	**−**	**−**	**−**	**+**	**−**	**+**	**−**	**+**	**−**	**−**
**BIM**	**−**	**−**	**+**	**−**	**+**	**−**	**+**	**−**	**+**	**−**	**−**
**BSM**	**−**	**−**	**+**	**−**	**+**	**−**	**+**	**−**	**+**	**−**	**−**
**BOM**	**−**	**−**	**+**	**−**	**+**	**−**	**+**	**−**	**+**	**−**	**−**
**GIJ**	**−**	**−**	**+**	**−**	**+**	**−**	**+**	**−**	**+**	**−**	**−**
**GSJ**	**−**	**−**	**−**	**−**	**+**	**−**	**+**	**−**	**+**	**−**	**−**
**GOJ**	**−**	**−**	**+**	**−**	**+**	**−**	**+**	**−**	**+**	**−**	**−**
**GIM**	**−**	**−**	**+**	**−**	**+**	**−**	**+**	**−**	**+**	**−**	**−**
**GSM**	**−**	**−**	**+**	**−**	**+**	**−**	**+**	**−**	**+**	**−**	**−**
**GOM**	**−**	**−**	**+**	**−**	**+**	**−**	**+**	**−**	**+**	**−**	**−**
**SIJ**	**−**	**−**	**−**	**−**	**+**	**−**	**+**	**−**	**+**	**−**	**−**
**SSJ**	**−**	**−**	**−**	**−**	**+**	**−**	**+**	**−**	**+**	**−**	**−**
**SOJ**	**−**	**−**	**−**	**−**	**+**	**−**	**+**	**−**	**+**	**−**	**−**
**SIM**	**−**	**−**	**−**	**−**	**+**	**−**	**+**	**−**	**+**	**−**	**−**
**SSM**	**−**	**−**	**−**	**−**	**+**	**−**	**+**	**−**	**+**	**−**	**−**
**SOM**	**−**	**−**	**−**	**−**	**+**	**−**	**−**	**−**	**−**	**−**	**−**
**VIJ**	**−**	**−**	**−**	**−**	**+**	**−**	**+**	**−**	**+**	**−**	**−**
**VSJ**	**−**	**−**	**−**	**−**	**+**	**−**	**+**	**−**	**+**	**−**	**−**
**VOJ**	**−**	**−**	**−**	**−**	**+**	**−**	**+**	**−**	**+**	**−**	**−**
**VIM**	**−**	**−**	**−**	**−**	**+**	**−**	**+**	**−**	**+**	**−**	**−**
**VSM**	**−**	**−**	**−**	**−**	**+**	**−**	**+**	**−**	**+**	**−**	**−**
**VOM**	**−**	**−**	**−**	**−**	**−**	**−**	**−**	**−**	**−**	**−**	**−**

First letter: V, Vik RA; B, Bore; G, Grødaland; S, SNJ; second letter: I, inlet; S, sludge; O, outlet; third letter: J, January; M, May. Red and Green coloring serves to emphasize the positive and negative result.

### 3.3 Microbial community

The sequencing data from the four WWTPs exhibited a Good’s coverage of at least 0.95 for all samples. The Shannon index was slightly higher for the May samples (5.9–9.8) than for the January samples (5.5–7.5). The number of OTUs over all samples ranged from 750 to 2,700. In general, the number of OTUs was higher in the sludge samples than in the inlet and outlet samples. The exceptions to this were the May samples from Vik RA and Mekjarvik (SNJ). The inlet and outlet samples for both WWTPs had numbers of OTUs at least 45% higher than those of the sludge samples. The precise Shannon and Chao1 values and the coverage can be found in **Supplementary Table S4**.

**Figure 1:**
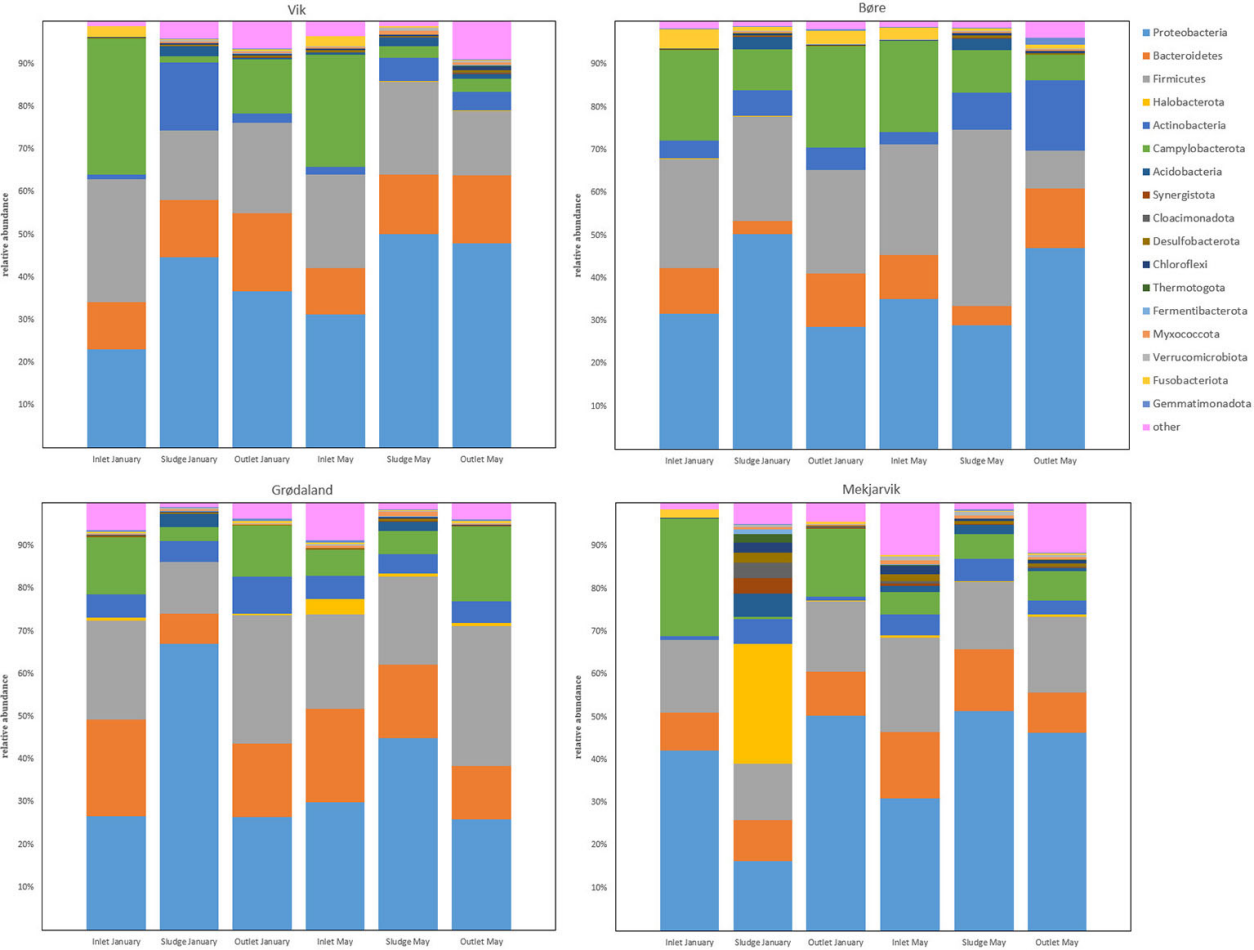
The common bacterial phyla with more than 1% relative abundance in at least one sample obtained. The legend indicates the color corresponding to each phylum.

**Figure 1** presents the most abundant phyla from the four WWTPs, Vik RA, Bore, Grødaland, and Mekjarvik, in January and May, with at least 1% relative abundance of the total population. The five most abundant phyla were Proteobacteria, Bacteroidetes, Firmicutes, Actinobacteria, and Campylobacterota, with varying relative abundance. In addition to the five most common phyla, Acidobacteria could be detected in all the sludge samples, with an average abundance of 2.8%. This phylum was also detected in the May inlet sample from Mekjarvik and in the outlet sample from Vik RA. The phylum also Fusobacteriota was present, with higher relative abundance in the inlet samples from Vik RA (4% and 3%) and Bore (2% and 2%) in January and May and from Mekjarvik (2%) in January. Fusobacteriota was also detected in the outlet samples with lower than 1% abundance, with the exception of Bore in January, with an abundance of 3%. The noticeable outlier was the January sample from Mekjarvik, where the most dominant phylum was Archaea Halobacterota, with 28% abundance. In addition, the phyla Synergistota, Cloacimonadota, Desulfobacterota, Chloroflexi, Chermotogota, and Fermentibacterota, were also present, with very low relative abundance in most samples. The aforementioned phyla appeared in proportions between 1% and 4% in the same sample from Mekjarvik.

The most common genera at Vik RA were *Acidovorax*, *Acinetobacter*, *Agitococcus*, *Arcobacter*, *Bacteroides*, *Flavobacterium*, *Lactococcus*, *Hydrogenophaga*, *Paludibacter*, *Pseudarcobacter*, *Rhodoferax*, and *Trichococcus*. The 13% proportion of *Acinetobacter* in the inlet samples (January and May) was relatively high, but its relative abundance fell in the sludge and the outlet water samples to 1% and 3% in January and 2% and 4% in May, respectively. *Agitococcus* was only detected at low levels (<0.2%) in the inlet and had a higher relative abundance in the outlet samples, at 2% in January and 6% in May. *Lactococcus* was present at 10% and 8% in the inlet in January and May, respectively, and its relative abundance was reduced to 2% and 1% in the outlet samples, respectively. *Rhodoferax* was present at 1% in the inlet, which increased to 10% and 8% in the sludge and 13% and 9% in the outlet in January and May, respectively.

At Bore, the most common genera were *Acidovorax*, *Acinetobacter*, *Aeromonas*, *Arcobacter*, *Lactococcus*, *Pseudarcobacter*, and *Trichococcus*. In January, *Acinetobacter* was present with a relative abundance of 18% in the inlet and 26% in the sludge, which decreased to 10% in the outlet. In May, the relative abundances were 23%, 6%, and 5% in the inlet, sludge, and outlet samples, respectively. These results indicate a large difference in the relative abundance of *Acinetobacter* in the sludge in January compared with May. *Lactococcus* exhibited a drastic reduction in the relative abundance between the sludge and outlet samples in May, in which the overall abundance fell from 13% in the sludge to 0.7% in the outlet. This decrease was not observed in January samples, where *Lactococcus* fell from 11% to 8%. *Leucobacter*, which was only present in the inlet samples at <2.2%, was present in the May outlet at 10%. Although the relative abundances of *Pseudarcobacter* in the inlet and sludge were comparable in the January and May samples, in the outlet samples, more variation was observed, with the abundance of *Pseudarcobacter* at 14% in January, decreasing to 3% in May.

**Figure 2:**
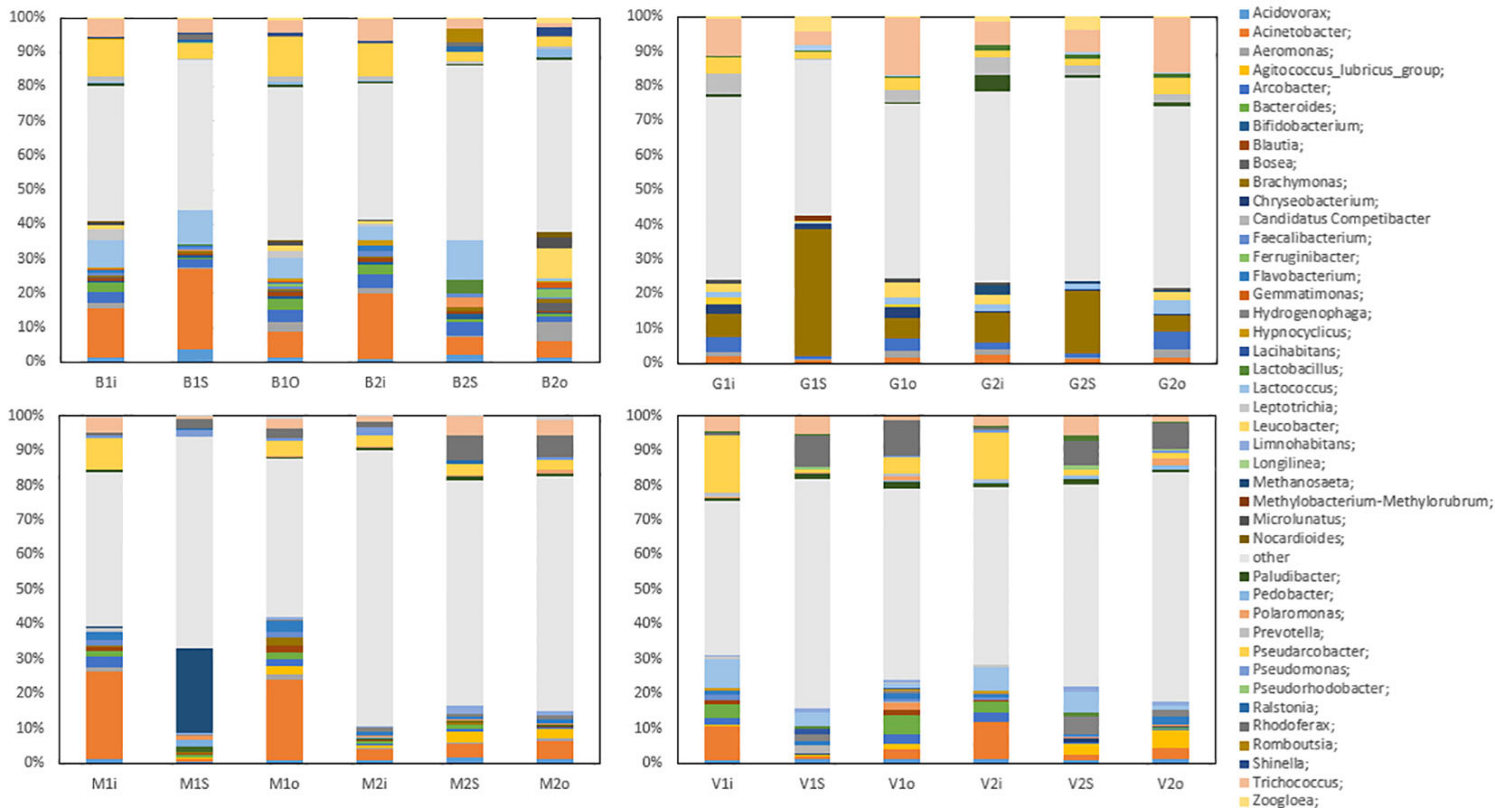
The common bacterial genera with more than 1% relative abundance in at least one sample obtained. The abbreviations B, G, S, and V represent the four WWTPs: Bore, Grødaland, SNJ, and Vik RA, respectively. The legend indicates the color corresponding to each genus.

**Figure 2** shows the most abundant bacterial genera from the four WWTPs Vik RA, Bore, Grødaland and Mekjarvik in January and May with at least 1% relative abundance of the total population. The most common genera found at Grødaland were *Acinetobacter*, *Aeromonas*, *Arcobacter*, *Brachymonas*, *Chryseobacterium*, *Lactococcus*, *Leucobacter*, *Prevotella*, *Pseudarcobacter*, *Trichococcus*, and *Zoogloea*. Compared with the other WWTPs, *Acinetobacter* comprised a lower proportion of all samples, at approximately 2% of the community. Another outlier was *Brachymonas*, which was present with an abundance >2.4% only in the Grødaland samples. The abundance of *Brachymonas* as a percentage of the total community decreased from 8% and 11% in January to 7% and 6% in May at Grødaland. Its proportion in the sludge was 40% in January and 21% in May. *Trichococcus* was also present in higher proportions at Grødaland compared with the other WWTPs, at 13% and 8% in January and May in the inlet, and exhibited a further increased in the outlet at 21% and 20%.

At Mekjarvik (SNJ), the most common genera were *Acinetobacter*, *Methanosaeta*, *Pseudarcobacter*, *Rhodoferax*, and *Trichococcus*. *Acinetobacter* was found in high proportions in the January samples, at 29% in the inlet and 26% in the outlet. The main outlier was the Archaea genus *Methanosaeta*, at 25% in the January sludge sample, corresponding to the Halobacterota phylum.

Of the pathogens from the ESKAPEE group (*Enterococcus faecium*, *S. aureus*, *Klebsiella pneumoniae*, *Acinetobacter baumannii*, *Pseudomonas aeruginosa*, *Enterobacter* spp., and *Escherichia coli*), only *Enterococcus faecium* and *E. coli* were detected. *E. faecium* was at less than 1% in all inlet and outlet samples (both months), and *E. coli* was at less than 1% in all inlet, outlet, and sludge samples (both months).

## 4 Discussion

To determine whether wastewater influents have an effect on the microbial community and resistance pattern, samples were collected from four WWTPs in Rogaland County, Norway, which received wastewater from areas with varying population densities and industries.

The OTU values (750–2,700) and the Shannon index (5.5–9.8) (**Supplementary Table S4**) indicated that the WWTPs investigated in this study contained a rich and diverse bacterial community. The literature on WWTP microbial communities reports OTU counts in the range of 750–970 and Shannon index values of 3.7–5.7, which are lower than those observed for the microbial communities in this study, demonstrating that the microbial communities of the WWTPs in this study were very diverse (Wolff et al., 2018; Do et al., 2019; Yang et al., 2020). The WWTPs at Mekjarvik (SNJ) and Vik RA, receiving municipal wastewater with higher population equivalents, had the highest OTUs (particularly in May).

Many of the ARGs that are commonly present, such as the β-lactamases *bla*_SHV-1_ and *bla*_TEM-1_ (Peirano and Pitout, 2019; Mirkalantari et al., 2020), were not detected at the four treatment plants in this study. Only the *msrA*, *ermB*, *tetA*, and *tetM* genes were detected via PCR assay. Exceptions to this were the outlet samples from Vik RA and Mekjarvik (SNJ) in May. In the Vik RA sample, no resistance genes were detected, and in the case of Mekjarvik (SNJ), only *ermB* was detected. For the most part, when these genes were present in the influent, they were not removed during the treatment process and were still present in the effluent, and were thus released into the surrounding environments. This was expected, as none of the four treatment plants was designed with the removal of ARGs in mind. Putting this into perspective, other Norwegian WWTPs have been found to have a higher prevalence of ampicillin resistance than WWTPs in the Rogaland region. In a study conducted in the Oslo region, *bla*_TEM-1_ and *tetM* were the only resistance genes found; this finding concurs with the low number of resistance genes found in this study, and both genes could be found in the influent and effluent of one Oslo-based WWTP (Cacace et al., 2019). Cacace et al. (2019) also investigated six additional ampicillin resistance genes, which were not tested in our study, and only *bla*_OXA-58_ was found to be present in higher numbers than *bla*_TEM-1_, with *tetM* numbers being comparable to those observed for *bla*_OXA-58_ in Oslo. This observation also applied to other European WWTPs tested in the study, such as in Germany and the Netherlands (Cacace et al., 2019). In Bergen, Radisic et al. (2023) examined *K. pneumoniae* at five different WWTPs and found that the most common forms of resistance among *K. pneumoniae* were against ampicillin and azithromycin. The most common ampicillin resistance genes originated from the extended-spectrum β-lactamase (ESBL) family CTX-M with several SHV and TEM representatives (Radisic et al., 2023), with the latter not being detected in Rogaland. Alexander et al. (2020) studied different WWTP clusters (communal, food industry, and hospital) and found that more than 99% of ARGs consisted of *ermB*, *tetM*, *bla*_TEM_, and *sul1*, with slightly higher abundances observed in hospital wastewater. The wastewater was also tested for *vanA*, which was categorized as rarely occurring (Alexander et al., 2020). These results correspond to our findings in terms of the presence of *ermB* and *tetM* in nearly all samples and the absence of *vanA*.

The MIC_50_ and MIC_90_ values indicated higher resistance to ampicillin and vancomycin. The ampicillin resistance observed in this study is very common, as ampicillin resistance occurs naturally in soil bacteria, and the number of variants for *bla*_SHV_ and *bla*_TEM_ combined exceeds 450 (NCBI, 2023a; NCBI, 2023b). As these were not found in our study, it can be assumed that the ampicillin resistance was conferred by a different resistance gene, for example, from the CTX-M gene family (Peirano and Pitout, 2019). As the two tested vancomycin resistance genes were not found in the samples, the high MIC values can be explained by either the presence of untested resistance genes or the presence of mostly gram-negative bacteria. Vancomycin’s main targets are gram-positive bacteria, and it has nearly no effect on gram-negative bacteria (Mühlberg et al., 2020). In Buenos Aires, Argentina, the MIC_50_ and MIC_90_ values were compared for ampicillin and vancomycin in hospital and municipal wastewater. For vancomycin, both values for both sampling sites exceeded 960 mg/L. Ampicillin in the municipality wastewater was also >960 mg/L, whereas in the hospital wastewater, the MIC_50_ value was 240 and the MIC_90_ value was 480 mg/L (Nuñez et al., 2016). Li et al. (2009) studied bacterial strains from a WWTP treating penicillin production wastewater. For ampicillin, the MIC_50_ and MIC_90_ values were >1,024 mg/L. For tetracycline, the MIC_50_ value was 8 mg/L and the MIC_90_ value was 512 mg/L (Li et al., 2009). MIC values for ampicillin are challenging to use as a point of comparison, as the values exceeded the established scale set by the experiment. In Norway, the measured values were lower for vancomycin at MIC_50_ and tetracycline at MIC_50_ and MIC_90_.

The low MIC values and the detection of only a small number of resistance genes indicate that the maintenance of human consumption of antibiotics below the European average, along with Norway being a country with one of the lowest levels in Europe of antibiotic consumption by food-producing animals, is a successful strategy for reducing the presence of antibiotic resistance genes in wastewater (European Centre for Disease Prevention and Control (ECDC), EFSA and EMA, 2017; European Centre for Disease Prevention and Control (ECDC), 2021). Comparing these results with those obtained in Germany, which has a comparable human development index and a lower human community use of antibiotics, but has considerably higher antibiotic use in food-producing animals (European Centre for Disease Prevention and Control (ECDC), EFSA and EMA, 2017; European Centre for Disease Prevention and Control (ECDC), 2021), certain similarities and differences are recognizable. Alexander et al. (2020) studied different-sized WWTPs in Germany with municipal, food, and hospital wastewater. They demonstrated that, independently of the catchment area, all WWTPs were contributing to the release of ARGs into the environment. The greatest difference was detected in hospital wastewater, where a stronger correlation between ARGs and facultative pathogenic bacteria was observed (Alexander et al., 2020). Pärnänen et al. (2019) examined ARGs in urban wastewater treatment plants (UWTPs) in different European countries *via* qPCR. The Norwegian UWTP (eastern Norway) exhibited very low numbers of AMP and VAN resistance genes in the inlet and outlet. This is consistent with our detection of no AMP or VAN resistance genes *via* PCR. They also concluded that antibiotic consumption had a lower impact than external environmental factors (e.g., temperature) (Pärnänen et al., 2019). This is also concordant with the findings of this study and the study by Alexander et al. (2020), with results indicating that the source of the wastewater did not influence the resistome strongly but rather that temperature is a controlling factor. MacFadden et al. (2018) demonstrated by studying three common bacterial pathogens (*E. coli*, *K. pneumoniae*, and *S. aureus*) in the USA that an average increase of 10°C can lead to an increase in ARGs in this population by up to 10%. McGough et al. (2020) demonstrated using the same bacterial pathogens (*E. coli*, *K. pneumoniae*, and *S. aureus*) that higher ambient temperatures favor antibiotic resistance in *E. coli* and *K. pneumoniae* (McGough et al., 2020).

### 4.1 Microbial analysis

Typical phylum compositions observed at WWTPs with different catchment areas in China, Canada, the USA, and Germany (Hu et al., 2012; Zhang et al., 2012; Ju et al., 2014; Wolff et al., 2018; Ma et al., 2023) are similar to the those observed for all four WWTPs in this work. This includes the five most common phyla, Proteobacteria, Firmicutes, Bacteroidetes, Campylobacter, and Actinobacteria, along with phyla such as Acidobacteria and Verrucomicrobia.

Fusobacteriota was found to comprise more than 1% of the OTUs detected in the inlet samples from the Bore, SNJ, and Vik RA WWTPs in January and May and was present in the outlet sample from Bore in May. The Fusobacteriota phylum has recently been found to be statistically associated with carbapenemase-encoding genes in hospital wastewater and its members have been identified as potential ARG reservoirs (Hubeny et al., 2021). Most of the fusobacteria detected in this study were determined to be composed of the pathogen *Leptotrichia*, which occurs in the human oral cavity, among other biomes (Eribe and Olsen, 2017). Quite recently, *Leptotrichia* has also been found in WWTPs in Finland and South Africa (Leiviskä and Risteelä, 2022; Poopedi et al., 2023). As the *Leptotrichia* genus can be involved in opportunistic infections through blood or oral transmission (Eribe and Olsen, 2017) and may be a reservoir for ARGs, it is therefore important for public health. *Leptotrichia* was reduced in the outlet on average from 1.2%–2.2% to <0.6% in SNJ and Vik RA. The percentage of *Leptotrichia* at the Bore WWTP was reduced from 3.7% to 1.8%. Bore, unlike the other WWTPs included in this study, does not include secondary treatment (**Supplementary Table S1**). Although Fusobacteriota has previously been found to be associated with common carbapenemases, such as *bla*_TEM_, this gene was not found at any of the WWTPs in this study.

One exception observed in the sequencing results when compared with the other three WWTPs in this study was the high number of the Archaea phylum Halobacterota found at the SNJ WWTP in January, comprising 28% of the total population. The largest part of the Halobacterota community was found to be composed of the genus *Methanosaeta*, comprising 25% of the total microbial community. *Methanosaeta* is normally found in anaerobic digestion reactors and in upflow anaerobic sludge blanket (UASB) reactors, as it is rather effective in adhesion and granulation (Liu and Whitman, 2008). We assume the reason that *Methanosaeta* represents such a high proportion of the microbial community at SNJ is the anaerobic digester return flow following sludge dewatering of digested sludge, hence leading to the presence of the Halobacterota phylum in the activated sludge despite the fact that the aerobic environment is not suited to this phylum.

The genus *Acinetobacter* has often been found to be associated with the removal of phosphate from wastewater and activated sludge (Oerther et al., 2002). At the same time, it includes species such as *A. baumannii*, *Acinetobacter calcoaceticus*, and *Acinetobacter lwoffii*, which have been described as highly virulent pathogens (Rosalino et al., 2020). These three species were not detected in the samples in this study, and *Acinetobacter johnsonii* comprised the majority of the *Acinetobacter* genus detected. *A. johnsonii* has been described as a bioaccumulator of phosphate (Boswell et al., 2001).

Detected ESKAPEE pathogens were limited to *E. faecium* and *E. coli*. Although many of these pathogens can grow in lower temperatures, the optimal growth temperatures are >30°C. Specifically, the optimal growth temperatures of these pathogens are as follows: *E. faecium*, 42°C (Fisher and Phillips, 2009); *S. aureus*, 30°C–37°C (Kadariya et al., 2014); *K. pneumoniae*, 30°C–35°C; *A. baumannii*, 33°C–35°C; *P. aeruginosa*, 37°C; and *E. coli*, 37°C (Garrity, 2007). As Stavanger (the administrative center of Rogaland County) had an average temperature of 4.3°C and 10°C in January and May, respectively (Norwegian Meteorological Institute, 2022), the low number of ESKAPEE microbes is to be expected. This ties in with the previously presented argument that higher temperatures are a major factor in high ARG and pathogen presence. A putative hypothetical explanation for this effect may be that cellular growth, rather than horizontal gene transfer, is the dominant mechanism in ARG spread. As pathogens, especially colon bacteria, are expected to have a stricter growth temperature range, low mesophilic temperate conditions may impose a strong selective pressure for non-pathogens, and thus, vertical spreading becomes limited. Theoretical modeling conducted in our group indicates that vertical ARG transfer is a determinative mechanism in high-growth environments such as nutrient-rich WWTPs (Stokka, 2024). Temperature may also affect HGT directly (e.g., via reduced competence or transfer dynamics), which is also a possible explanation. This aspect of how temperature affects the spread of ARGs should be further investigated in controlled growth and gene transfer studies.

## 5 Conclusion and outlook

The results presented here demonstrate that, overall, differences in the presence of ARGs and in MIC values are only slightly influenced by catchment area activities, wastewater sources, and/or treatment process configuration. The same is true for the different microbial community compositions, which do not deviate significantly in Norway compared with communities observed in studies from other countries around the world. It can be observed that the resistance genes that were present were not removed from the wastewater, meaning that additional unit treatment processes are required for active removal. On the basis of the same comparative observations, we hypothesize that environmental factors (e.g., temperature) influence the resistome by vertical rather than horizontal gene transfer to a larger degree than antibiotic concentrations or wastewater origin.

## Data availability statement

The datasets presented in this study can be found in online repositories. The names of the repository/repositories and accession number(s) can be found below: https://www.ebi.ac.uk/ena/browser/view/PRJEB70859.

## Author contributions

DB: Conceptualization, Investigation, Methodology, Writing – original draft, Writing – review & editing. RK: Data curation, Funding acquisition, Methodology, Writing – review & editing. KK: Conceptualization, Methodology, Supervision, Writing – review & editing.
