# Supplementary material for: The presence of antibiotic-resistant bacteria at four Norwegian wastewater treatment plants: seasonal and wastewater-source effects
Source: Front Antibiot. 2024 Feb 7;3:1351999. doi: 10.3389/frabi.2024.1351999 (PMC11731629; doi:10.3389/frabi.2024.1351999)
Supplement: Supplementary file 1 [file DataSheet_1.docx]

**Supplementary Material**

Table S1: Key figures describing the loading and composition of wastewater and the size, sources and catchment area activities for the different WWTP process configurations. Composition and loadings are annual averages (2022) and variability (std. dev.) based on 48, 28, 23, and 26 weekly and bi-weekly daily composite samples (flow proportional) taken from the inlet of SNJ, Vik RA, Grødaland and Bore WWTP, respectively.

Table S2: The colony forming units (CFU) for the sludge samples collected per gram sludge.

|  | **January (cfu/g x10^6^)** | **May (cfu/g x10^6^)** |
| --- | --- | --- |
| **Vik** | 9.9 ± 0.25 | 8.7 ±0.19 |
| **Bore** | 8.1 ± 0.23 | 8.3 ± 0.5 |
| **Grødaland** | 8.8 ± 0.1 | 9.1 ± 0.14 |
| **Mekjarvik** | 9.5 ± 0.14 | 9.9 ± 0.2 |

Table S3: The MIC values for the 24 randomly selected strains of each sample and date.

| **AMP** | **January** | | | | **May** | | | |
| --- | --- | --- | --- | --- | --- | --- | --- | --- |
|  | **Vik** | **Bore** | **Grødaland** | **Mekjarvik** | **Vik** | **Bore** | **Grødaland** | **Mekjarvik** |
| 1 | 8 | >128 | >128 | <0.25 | 128 | 128 | 32 | >128 |
| 2 | 0.5 | >128 | 16 | 32 | 16 | 64 | 128 | >128 |
| 3 | >128 | >128 | >128 | 32 | 128 | 32 | 32 | >128 |
| 4 | >128 | >128 | >128 | >128 | >128 | >128 |  | >128 |
| 5 | 128 | >128 | 2 | >128 | >128 | >128 | 16 | >128 |
| 6 | >128 | >128 |  | >128 | 4 | >128 | 8 | >128 |
| 7 | 32 | >128 | >128 | >128 | >128 | 16 | 32 | >128 |
| 8 | >128 | >128 | >128 | >128 | >128 | 4 | 2 | >128 |
| 9 | >128 | 128 | >128 | >128 | >128 | 16 | 128 | 128 |
| 10 | >128 | >128 | >128 | >128 | >128 | >128 | 128 | >128 |
| 11 | 8 | 16 | >128 | >128 | 32 |  | >128 | >128 |
| 12 | >128 | >128 | >128 | >128 | >128 | >128 | >128 | >128 |
| 13 | 128 | 32 | >128 | >128 | 128 | 4 | >128 | >128 |
| 14 | 32 | 64 | 16 | 32 | 128 | 1 | 16 | >128 |
| 15 | 64 | >128 | >128 | >128 | >128 | 128 | 64 | >128 |
| 16 | 32 | >128 | >128 | 64 | >128 | 0.5 | 0.5 | >128 |
| 17 | >128 | 64 | >128 | >128 | >128 | 2 | >128 | 64 |
| 18 | >128 | >128 | 128 | 16 | >128 |  | 128 | >128 |
| 19 | >128 | >128 | 128 | >128 | >128 | 128 | 128 | >128 |
| 20 | >128 | 128 | >128 | 32 | >128 | 32 | >128 | >128 |
| 21 | 32 | >128 | >128 | >128 | >128 | >128 | <0.25 | >128 |
| 22 | >128 | <0.25 | >128 | >128 | >128 |  | >128 | >128 |
| 23 | 32 | <0.25 | >128 | >128 | 32 | >128 | 64 | >128 |
| 24 | >128 | <0.25 | 128 | >128 | >128 | 4 | 64 | >128 |
| **Tet** | **January** | | | | **May** | | | |
|  | **Vik** | **Bore** | **Grødaland** | **Mekjarvik** | **Vik** | **Bore** | **Grødaland** | **Mekjarvik** |
| 1 | 32 | 128 | 1 | 0.5 | <0.25 | 2 | 0.5 | 4 |
| 2 | <0.25 | 4 | 1 | 1 | 2 | 8 | 1 | 4 |
| 3 | 0.5 | 1 | 2 | 16 | <0.25 | 4 | 0.5 | 4 |
| 4 | <0.25 | 1 | 1 | 1 | 1 | 2 |  | 16 |
| 5 | 4 | 2 | <0.25 | 4 | 0.5 | 1 | 2 | 2 |
| 6 | <0.25 | 2 |  | 1 | 128 | 2 | 64 | 4 |
| 7 | <0.25 | 32 | 8 | 4 | 16 | 4 | 2 | 4 |
| 8 | 2 | 0.5 | 1 | <0.25 | 16 | <0.25 | 1 | 4 |
| 9 | >128 | <0.25 | 2 | 1 | 16 | 0.5 | 2 | 4 |
| 10 | 1 | 8 | 0.5 | 4 | 1 | 2 | 2 | 2 |
| 11 | 2 | 32 | 0.5 | <0.25 | 2 |  | 0.5 | 16 |
| 12 | 0.5 | 0.5 | 1 | 1 | 1 | <0.25 | 0.5 | 16 |
| 13 | <0.25 | 2 | 16 | 1 | 0.5 | 1 | 16 | 4 |
| 14 | <0.25 | 64 | 64 | 2 | 1 | 0.25 | 2 | 4 |
| 15 | 4 | 0.5 | <0.25 | 0.5 | 1 | 2 | 0.5 | 4 |
| 16 | 0.5 | 1 | 4 | 2 | 0.5 | <0.25 | <0.25 | 8 |
| 17 | 1 | 2 | <0.25 | 1 | 2 | <0.25 | 0.25 | 2 |
| 18 | <0.25 | 0.5 | 0.5 | 2 | 4 |  | 1 | 1 |
| 19 | 1 | 2 | 0.5 | >128 | 1 | 4 | 1 | 4 |
| 20 | 4 | 0.5 | 16 | 64 | 1 | 2 | 0.5 | 2 |
| 21 | <0.25 | 2 | 4 | 128 | 1 | 2 | 128 | 8 |
| 22 | 8 | 1 | 4 | 8 | 1 |  | 0.5 | 2 |
| 23 | <0.25 | 1 | 4 | 32 | 32 | 0.5 | 0.5 | 4 |
| 24 | 0.5 | <0.25 | 1 | 16 | 1 | <0.25 | 64 | 2 |
| **Van** | **January** | | | | **May** | | | |
|  | **Vik** | **Bore** | **Grødaland** | **Mekjarvik** | **Vik** | **Bore** | **Grødaland** | **Mekjarvik** |
| 1 | 32 | 16 | >128 | 16 | 128 | >128 | 32 | 128 |
| 2 | 16 | >128 | 8 | 16 | 128 | >128 | 64 | >128 |
| 3 | 32 | 32 | 128 | 32 | 128 | 16 | 64 | 128 |
| 4 | 64 | 32 | >128 | >128 | 128 | 128 |  | 128 |
| 5 | 16 | 128 | 8 | 128 | 128 | 128 | 16 | 128 |
| 6 | 32 | 64 |  | 64 | 32 | 64 | >128 | 128 |
| 7 | 8 | 16 | 8 | 64 | 8 | 128 | 32 | 128 |
| 8 | 32 | 64 | 32 | 8 | >128 | <0.25 | >128 | 128 |
| 9 | 16 | 128 | 128 | 32 | 64 | <0.25 | 64 | 64 |
| 10 | >128 | 128 | 64 | >128 | 128 | 64 | >128 | 64 |
| 11 | 64 | >128 | 32 | <0.25 | 32 |  | >128 | 128 |
| 12 | 128 | 128 | 128 | 32 | >128 | >128 | >128 | 128 |
| 13 | 16 | 32 | 8 | 32 | 64 | 32 | 64 | 128 |
| 14 | 32 | 32 | 32 | 32 | 128 | 0.25 | 128 | 128 |
| 15 | 64 | >128 | 64 | 8 | >128 | 128 | 16 | 128 |
| 16 | 64 | >128 | >128 | 64 | >128 | <0.25 | <0.25 | 128 |
| 17 | 32 | 128 | 16 | 2 | >128 | <0.25 | 32 | >128 |
| 18 | 32 | 64 | 64 | 32 | 64 |  | 128 | 128 |
| 19 | 16 | 16 | 16 | 16 | >128 | >128 | 64 | >128 |
| 20 | 8 | 32 | 32 | 16 | 64 | >128 | 64 | 128 |
| 21 | 128 | >128 | 16 | 128 | 128 | >128 | 32 | 128 |
| 22 | >128 | 2 | 64 | 8 | 128 |  | >128 | 128 |
| 23 | 8 | 32 | >128 | 32 | 32 | 32 | 64 | 128 |
| 24 | >128 | 32 | 8 | 16 | >128 | <0.25 | 64 | 32 |

Table S4: OTU, Shannon index, chao1 and Goods Coverage for the four WWTPs. B=Bore, G= Grødaland, M= Mekjarvik, V= Vik RA, i=inlet, S=sludge, o=outlet, 1=January and 2= May.

| Sample | OTU | Shannon index | chao1 | Goods coverage |
| --- | --- | --- | --- | --- |
| B1i | 852 | 6.309 | 1162.257 | 0.985 |
| B1S | 1178 | 6.666 | 1615.215 | 0.977 |
| B1O | 924 | 6.709 | 1387.76 | 0.983 |
| B2i | 829 | 6.226 | 1142.851 | 0.985 |
| B2S | 1374 | 7.569 | 1664.51 | 0.977 |
| B2o | 894 | 6.891 | 1098.455 | 0.988 |
| G1i | 949 | 6.572 | 1354.399 | 0.983 |
| G1S | 1128 | 5.581 | 1471.727 | 0.978 |
| G1o | 839 | 6.039 | 1052.293 | 0.987 |
| G2i | 1012 | 6.929 | 1256.722 | 0.985 |
| G2S | 1366 | 7 | 1802.65 | 0.975 |
| G2o | 852 | 6.086 | 1119.534 | 0.986 |
| M1i | 870 | 6.08 | 1281.444 | 0.982 |
| M1S | 1166 | 6.815 | 1515.38 | 0.979 |
| M1o | 1018 | 6.601 | 1578.048 | 0.978 |
| M2i | 2712 | 9.859 | 3450.092 | 0.956 |
| M2S | 1345 | 7.895 | 1664.132 | 0.977 |
| M2o | 2049 | 8.602 | 2721.308 | 0.962 |
| V1i | 755 | 5.948 | 1055.805 | 0.986 |
| V1S | 1168 | 7.12 | 1591.073 | 0.977 |
| V1o | 1659 | 7.875 | 2524.786 | 0.964 |
| V2i | 1600 | 7.004 | 2325.113 | 0.966 |
| V2S | 1162 | 7.386 | 1472.004 | 0.98 |
| V2o | 1770 | 8.469 | 2134.669 | 0.973 |

Table S5: The TSS and VSS values from the four different WWTPs for inlet and outlet in January and May.

|  | January | | | | May | | | |
| --- | --- | --- | --- | --- | --- | --- | --- | --- |
|  | TSS | | VSS | | TSS | | VSS | |
|  | Inlet | Outlet | Inlet | Outlet | Inlet | Outlet | Inlet | Outlet |
| Vik | 250.00 | 33.91 | 232.75 | 33.55 | 391 | 28.7 | 378.5 | 27 |
| Bore | 199.00 | 128.17 | 198.25 | 114.00 | 341.33 | 151.5 | 298.33 | 135.5 |
| Grødaland | 772.08 | 75.90 | 679.17 | 64.12 | 626 | 290.67 | 572 | 265.67 |
| Mekjarvik | 207.07 | 21.00 | 185.37 | 20.17 | 187.5 | 18.1 | 161.25 | 16.6 |


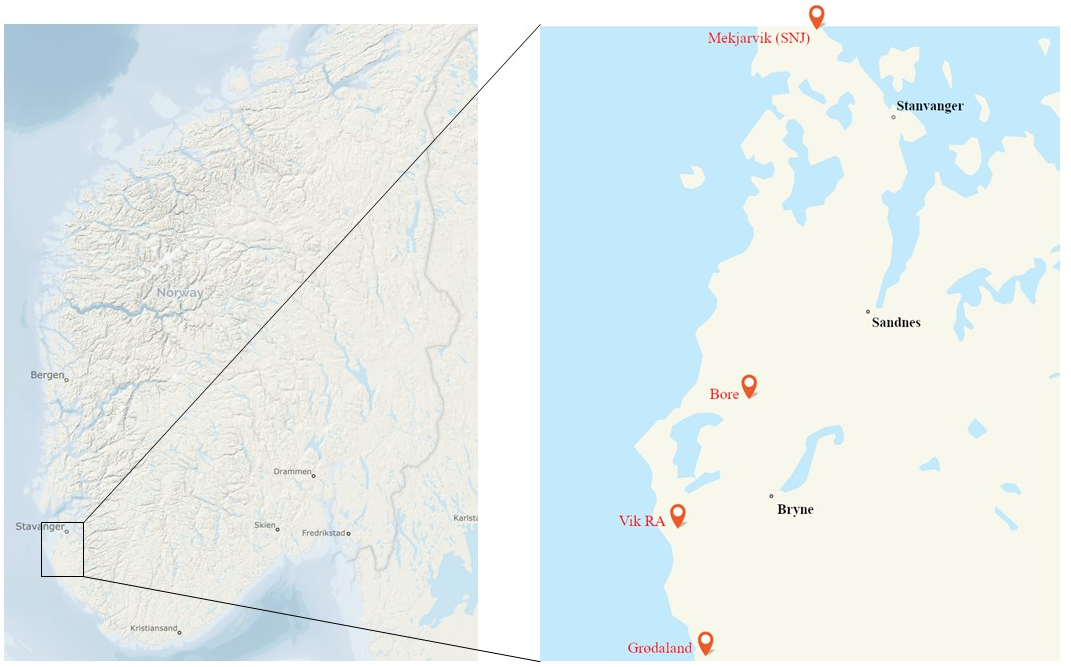


Figure S1: Locations of the four WWTPs in Rogaland, Norway.
